# Examination of Sex-Related Differences in Fatigability and Frequency Components of Mechanomyographic Signals During Sustained Exercise

**DOI:** 10.3390/muscles3040035

**Published:** 2024-12-04

**Authors:** Brian Benitez, Minyoung Kwak, Pasquale J. Succi, Clara J. Mitchinson, Haley C. Bergstrom

**Affiliations:** Department of Kinesiology and Health Promotion, University of Kentucky, Lexington, KY 40536, USA; minyoung.kwak@uky.edu (M.K.); psu236@g.uky.edu (P.J.S.); clara.mitchinson@uky.edu (C.J.M.)

**Keywords:** sex-differences, neuromuscular-responses, wavelet-analysis, mechanomyography

## Abstract

Background: Surface mechanomyographic (sMMG) signals have been used to examine sex-specific differences in the mechanical behavior of muscle during fatiguing exercise. However, studies often utilize simple amplitude- and frequency-based analyses, which only reveal the static components of the sMMG signal. Methods: Thus, a wavelet-based analysis was used to examine changes in the spectral intensity of the non-dominant limb’s vastus lateralis during a fatiguing, maximal, unilateral isometric leg extension in recreationally active men (*n* = 11) and women (*n* = 10). Relative changes in spectral intensities and instantaneous mean frequency (IMF) were examined using linear mixed-effect models. Time-to-task failure was compared using an independent sample *t*-test. Results: The neuromuscular responses demonstrated parallel decreases in IMF (*p* < 0.001). Further, there were parallel, nonlinear, decreases in spectral intensity across wavelets (*p* < 0.001) and there were no sex differences in time-to-task failure (*p* = 0.15). Conclusions: These data showed no sex-specific differences in exercise fatigability or muscle mechanics during fatiguing exercise of the leg extensors. However, when collapsed across sex, wavelet-specific changes in spectral intensity over time reveal novel insights into the interplay between low- and high-frequency components during fatigue.

## 1. Introduction

The surface mechanomyograhic (sMMG) signal records and quantifies the low-frequency lateral oscillations of active skeletal muscle fibers [1,2], which has been previously used to examine the mechanical behavior of muscle [3,4,5]. Although the exact origin(s) of the sMMG signal remains elusive, Gordon and Holbourn [6] have suggested that these oscillations reflect the “mechanical counterpart” of the motor unit’s electrical activity as measured by surface electromyography (sEMG). Furthermore, Barry and Cole [7] and Orizio [8] have suggested that the lateral oscillations recorded by the sMMG signal are a function of (1) a gross lateral movement at the initiation of a contraction generated by the non-simultaneous activation of muscle fibers, (2) smaller subsequent lateral oscillations generated at the resonant frequency of the muscle, and (3) dimensional changes of the active fibers.

Using the amplitude and frequency characteristics of the sMMG signal, previous research has been able to qualitatively examine motor control strategies across various resistance exercise applications, including isometric [9,10,11], isotonic (also known as dynamic constant external resistance) [12], and isokinetic [13,14] muscle actions. The previous literature has suggested that the frequency characteristics of the sMMG signal may provide qualitative information regarding the global firing rates of the unfused activated motor units [1,2]. Furthermore, the amplitude characteristics of the sMMG signal have been shown to be linked with the number of active motor units during electrically stimulated isometric contractions of isolated cat gastrocnemius muscle [10]. Thus, it has been suggested that sMMG amplitude may be an indicator of motor unit recruitment [2].

Despite its use for qualitatively assessing motor unit recruitment and firing rate modulation, the sMMG signal is often analyzed using traditional amplitude- and frequency-based methods that focus on static characteristics, overlooking its dynamic changes over time. For instance, while the Fast-Fourier Transform provides a detailed frequency spectrum of the sMMG signal [15], analyses often reduce this information to summary measures like mean or median frequency, which may obscure the nuanced evolution of frequency components during exercise [3,16,17,18]. To further our understanding of the etiology of any shifts in the frequency components of a signal, time–frequency signal processing techniques such as wavelet transform have been previously proposed [15,19]. In short, the wavelet analysis quantifies the intensity of the signal within defined frequency bands, enabling a finer-grained assessment of the interplay between low- and high-frequency components and their temporal changes. Originally developed for analyzing ‘events’ that produce non-stationary sEMG signals, Von Tscharner’s [20] wavelet-based time–frequency analysis utilizes a specialized set of non-linearly scaled Cauchy wavelets, tuned to the physiological response times of muscle twitches. Prior research demonstrates that the instantaneous frequencies derived from wavelet-based decomposition of sEMG signals can indicate the activity of different muscle fiber types during exercise [21]. Several studies have adapted similar wavelet-based techniques to the sMMG signal [22,23,24,25], providing an analysis of signal intensity that describes the power of a stochastic signal as a function of time and frequency. The results of these studies suggest that the wavelet-based decomposition of the sMMG signal may be particularly useful in studying fatigue during exercise, potentially offering more granular insights into the interplay between motor units of varying thresholds.

The study of fatigue during resistance exercise is a critical area of interest for exercise physiologists, as understanding the nuances of fatigue can aid in unraveling the complex neuromuscular dynamics that dictate human motor behavior [26]. Accordingly, the wavelet-based analysis of sEMG and sMMG signals has emerged as an invaluable tool in this field, offering greater insights into the temporal characteristics of motor unit properties as fatigue develops. One promising application of wavelet-based analysis is in investigating sex differences in resistance exercise fatigability, which have been partially attributed to variations in neuromuscular responses [27]. In fact, it is well documented that men and women differ in both anatomy and physiology, which typically results in marked sex differences in exercise fatigability. Men generally possess greater skeletal muscle mass than women, often accompanied by a higher proportion of fast-twitch muscle fibers [28], which contribute to superior strength and power capabilities [27,29]. Conversely, women tend to exhibit lower fatigability than men during sustained contractions at equivalent relative intensities [27,29]. Despite substantial evidence highlighting these sex-specific differences in exercise fatigability, the mechanisms driving these outcomes remain inadequately explained.

One of the primary determinants of exercise fatigability in both sexes is posited to be the specific site within the neuromuscular system that incurs the greatest stress, which varies depending on the task’s specific demands [27,29]. This differential stress influences the rate of fatigue development at particular neuromuscular sites, potentially resulting in sex-specific patterns of fatigability. However, evidence from neuromuscular research using sMMG-derived parameters remains inconsistent. For example, Nonaka et al. [18] observed sex differences in the sMMG–force relationship during isometric ramp contractions of the biceps brachii, attributing them to a predominance of slow-twitch motor unit activity and more pronounced fused tetanus in women. The men, however, exhibited higher relative mean frequencies as a function of relative torque. In contrast, Hill et al. [3] found no sex-related differences in sMMG amplitude or mean frequency during maximal voluntary isometric contractions (MVICs) or fatiguing intermittent submaximal (65% MVIC) isometric forearm flexion contractions. Given these conflicting findings, a more detailed analysis of the interplay between low- and high-frequency components of sMMG signals may reveal subtle nuances in neuromuscular responses that broader assessments might overlook. For instance, Murphy et al. [19] utilized wavelet-based analysis to investigate the electrophysiological characteristics of sEMG signals in the quadriceps during high- (70% MVIC) and low- (30% MVIC) torque isometric leg extensions. Despite similar mean frequencies across conditions, individual wavelet frequency components revealed distinct patterns: spectral intensity increased across all frequency domains during low-torque conditions, whereas in high-torque conditions, it increased in the lower-frequency domains but decreased in the higher-frequency domains. These findings highlight how similar aggregate signal properties can emerge from divergent neuromuscular adaptations, emphasizing the utility of further exploration beyond aggregated frequency responses. By applying wavelet-based analysis to sMMG signals, researchers may gain deeper insights into the neuromuscular mechanisms driving sex-specific differences in exercise fatigability.

The primary aim of this study was to examine underlying sex-specific differences in exercise fatigability by employing wavelet-based analysis of sMMG signals from the vastus lateralis during sustained, maximal, unilateral isometric leg extensions. While maximal, continuous exercise conditions are less frequently studied, they are crucial for elucidating the dose–response relationship between exercise intensity and performance-related fatigability. To address this objective, the study examined both shifts in mean frequency and the underlying frequency components contributing to the mean frequency. It was hypothesized that (1) sex-related differences would be observed in the shift of mean frequency and (2) sex-dependent differences would occur in the shifts of the underlying frequency components of the sMMG signal. These hypotheses are rooted in the known structural differences in muscle characteristics between sexes [28], which are expected to influence neuromuscular signal behavior. Despite conflicting evidence in previous sMMG studies, established distinctions in muscle fiber composition and size between men and women suggest that these structural differences are likely to manifest as variations in signal patterns during fatiguing exercise. Furthermore, it was hypothesized that women would exhibit greater fatigue resistance than men, consistent with extensive literature documenting women’s higher resistance to fatigue during sustained exercise [27,29].

## 2. Results

### 2.1. Change in Instantaneous Mean Frequency

**Figure 1:**
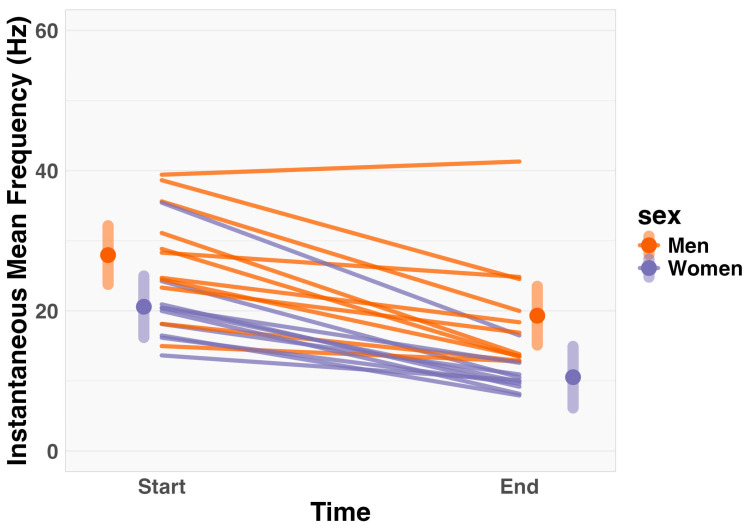
Change in instantaneous mean frequency (Hz) from start to end of task, separated by sex. Data are illustrated using estimated marginal means (large dots), 95% confidence intervals (error bars), and individual data (lines). Statistical analysis indicated there was no significant two-way interaction between sex and time. However, there was a main effect of time and sex, respectively.

Linear mixed-effects models showed no significant interaction between sex and time (F_1,19_ = 0.34, *p* = 0.57). However, there was a significant main effect of time (F_1,19_ = 60.59, *p* < 0.01), indicating a parallel decrease in IMF from the start to the end of the fatiguing task (Mean Difference [MD] = −9.35 Hz, 95% confidence interval [CI_95%_] = −11.86 to −6.83; Figure 1). Additionally, a significant main effect of sex was observed (F_1,26_ = 6.24, *p* = 0.02), showing that overall IMF differed between men and women (MD = −8.08 Hz, CI_95%_: −13.72 to −2.43). Specifically, men exhibited a significantly greater global IMF compared to women, with estimated marginal means of 23.62 Hz (CI_95%_ = 19.73 to 27.52) for men and 15.53 Hz (CI_95%_ = 11.47 to 19.64) for women.

### 2.2. Change in Spectral Intensity Across Wavelet Domains

Linear mixed-effects models revealed no significant three-way interaction between sex, wavelet domain, and time (F_2,332_ = 2.10, *p* = 0.12). However, a significant two-way interaction was observed between the wavelet domain and time (F_2,332_ = 63.25, *p* < 0.01), indicating parallel changes in spectral intensity over time across the wavelet domains for both men and women.

**Figure 2:**
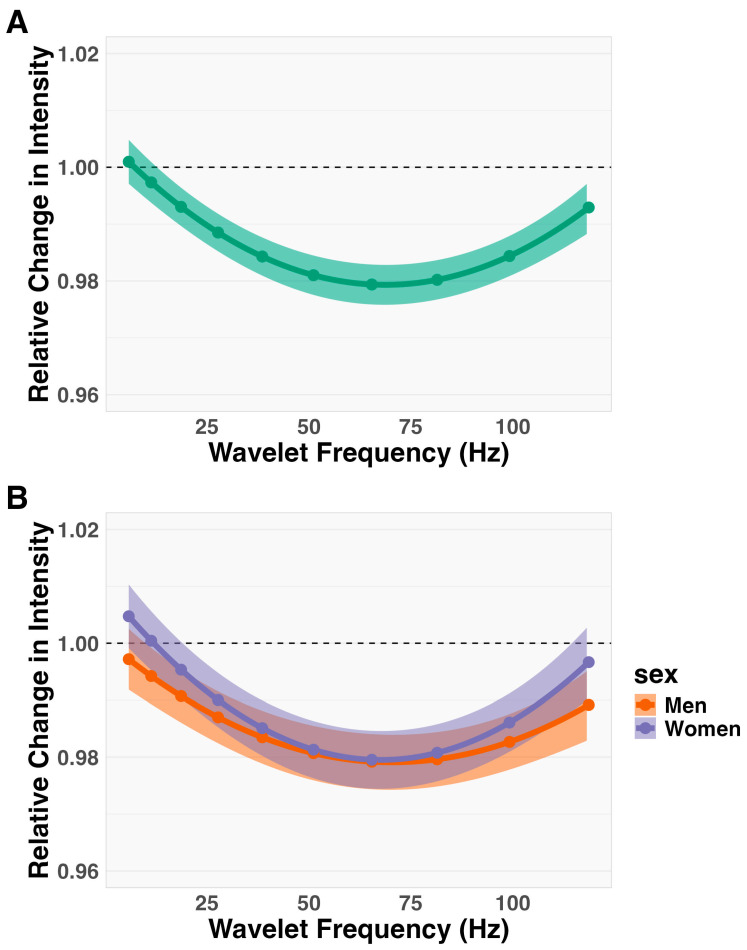
Relative change in spectral intensity plotted as a function of trial duration. The relationship is illustrated using model-predicted slopes (dots) and 95% confidence intervals (ribbons). To illustrate a null-effect while on the relative scale, a dashed line is presented at 1 on the y-intercept to represent 0 (i.e., no change in the relative scale). Statistical analysis indicated there was no significant three-way interaction between sex, time, and wavelet. This null effect is visibly demonstrated in the bottom subfigure. There was, however, a significant two-way interaction between wavelet and time, illustrated by the top subfigure (**A**). Furthermore, there was a main effect of sex, which can be identified in the bottom subfigure (**B**).

To explore the nature of this interaction, the relationship between relative change in frequency and trial duration was analyzed. Log-transformed intensity (log(I_k_)) was plotted against trial duration (Figure 2). When data were pooled across sex (Figure 2A), the lower frequency components showed no change in signal intensity over time. In contrast, the medium- to higher-frequency components exhibited a decrease in intensity, with the most substantial decline occurring in the medium frequencies.

Additionally, a significant main effect of sex was found (F_1,19_ = 49.49, *p* < 0.01), indicating a significant difference in the magnitude of spectral intensity between men and women. Back-transformed intensity values from the logarithmic scale revealed a 5.68-fold greater spectral intensity for men compared to women (CI_95%_ = 3.06 to 10.56).

### 2.3. Time-to-Task Failure

**Figure 3:**
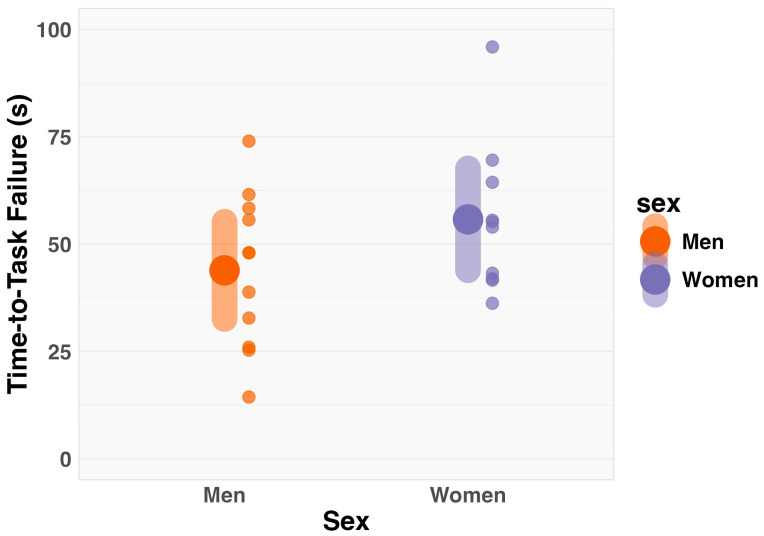
Comparison of time-to-task failure between men and women illustrated using estimated marginal means (large dots), 95% confidence intervals (error bars), and individual data points (small dots). Statistical analysis indicated no significant difference in time-to-task failure between men and women.

An independent sample *t*-test revealed no significant differences in time-to-task failure between men and women (MD = −11.87 s, CI_95%_ = −28.25 to 4.51; t_19_ = −1.52, *p* = 0.15; Figure 3).

## 3. Discussion

This was the first study to examine the sex-dependent frequency characteristics of the quadriceps using a wavelet-based analysis of sMMG. Contrary to our hypothesis, there were no significant sex-related differences in time-to-task failure to indicate that women were more fatigue-resistant than men. Further, there were no significant sex-related differences in the change in sMMG IMF, or spectral intensity within each wavelet domain. Although unrelated to our initial hypothesis, some noteworthy findings from the present study were that, for both men and women, there were nonlinear changes in the intensity spectra within each wavelet domain. Moreover, the magnitude of spectral intensity differed significantly between men and women, which indicated a greater overall spectral intensity for men compared to women. This significant difference in magnitude was also seen in the IMF analysis, which indicated overall higher frequencies for men compared to women.

The absence of sex-related differences in time-to-task failure is quite interesting. Generally, men have been shown to be more fatigable than women across various muscle groups [27], during both sustained and intermittent isometric tasks [30,31,32]. Specifically, for sustained, fatiguing tasks of the leg extensors, women are reportedly more fatigue-resistant than men across a variety of loading conditions [27]. Physiological differences, such as a greater proportion of slow-twitch fibers in women [28], have been proposed to explain this advantage. For instance, the higher proportion of slow-twitch fibers is associated with slower sarcoplasmic reticulum Ca^2^-ATPase activity and a slower rate of muscle relaxation [33], potentially granting women an edge during endurance-type tasks. In addition to fiber-type distribution, sex-related hormonal differences, such as the effects of estradiol, may also contribute to greater fatigue resistance in women. Estradiol reduces norepinephrine-induced vasoconstriction and enhances nitric oxide bioavailability [34], promoting a vasodilatory state and reducing vasoconstriction in nonactive vascular beds. This vasodilatory effect may improve blood flow to active muscles, supporting endurance. Furthermore, lower norepinephrine levels in women at rest and during submaximal exercise may limit cardiac contractility, reducing cardiovascular strain during prolonged activity [34]. Collectively, these factors have been proposed as explanations for the sex-related advantage observed in women when sustaining performance during endurance tasks involving the leg extensors. However, this advantage may diminish under heavier relative loading conditions. Previous research by Hunter [27] has shown a negative relationship between the intensity of sustained isometric contractions and sex differences, suggesting that as the relative force of the fatiguing task increases, sex-related differences in fatigue resistance decrease. This may be attributed to the larger absolute forces generated by men, particularly during low-force sustained isometric contractions, which result in greater intramuscular pressure and reduced blood flow compared to women [31,35]. With relatively high-force (>50%) isometric contractions, however, blood flow is more likely to be occluded in both men and women, reducing differences in perfusion and time-to-task failure [36]. Thus, it is plausible that during the fatiguing trials, force was sufficiently high to limit oxygen delivery similarly in both sexes, although this remains speculative without direct measurements of oxygenation.

Although sex did not participate in any interactions, the effect of sex for spectral intensity and IMF in the present study is not in and of itself surprising. An important consideration when interpreting the results of the present study is that sMMG signals are strongly influenced by factors such as muscle architecture [37], muscle stiffness [7], fiber type composition [7], subcutaneous adipose tissue [13], and intramuscular fluid pressure [38], all of which may differ between men and women. For example, and as previously stated, for the vastus lateralis, women have been shown to have a greater proportion of slow-twitch, oxidative, fibers [28]. Further, women have also been reported to exhibit greater relative fascicle lengths (relative to limb length) of the vastus lateralis and greater subcutaneous adipose tissue thickness in comparison to men [39]. Although not directly measured or quantified in the present study, a combination of these architectural and functional characteristics may have contributed to the observed global differences in spectral intensity and IMF between men and women.

Most of the previous literature investigating sMMG responses of the superficial quadriceps muscle has typically focused on a single parameter, such as mean amplitude or frequency [3,16,17,18]. However, the frequency component of the sMMG signal is inherently multifaceted, comprising multiple frequencies that represent diverse physiological inputs, including contributions from motor units across a spectrum of activation thresholds [1,2,15]. Consequently, these various inputs can converge to produce identical mean composite values, potentially masking the intricate motor control strategies utilized during exercise. In fact, using a similar wavelet-based analysis, Murphy et al. [19] recently demonstrated that convergence in the mean frequency components of the sEMG signal can occur via divergent signal adaptations. Such observations highlight the limitations of traditional frequency measurements in fully capturing the dynamic interplay of physiological factors influencing muscle function.

Although parameters derived from sEMG signals are reported more commonly in the literature, other studies have successfully utilized wavelet-based analysis of sMMG signals to decompose these signals into multi-resolution components [22,23,24,25]. For example, Beck et al. [24] employed wavelet analysis to examine the sMMG responses of quadriceps muscles during isometric leg extension muscle actions at intensities ranging from 20% to 100% of MVIC. Their findings revealed muscle-specific differences in spectral intensity across the range of relative intensities tested, showcasing the capability of wavelet analysis to detail variations in total sMMG intensity. Similarly, Armstrong [23] utilized a comparable wavelet technique—opting to use Morlet wavelets over the Cauchy wavelets used by Von Tscharner [20]—to explore the effects of fatigue and assess postural control during the single-legged stance, demonstrating the utility of the wavelet-based decomposition of sMMG signals in the study of fatigue and motor function. Our research builds on these prior studies, and the recent techniques employed by Murphy et al. [19], by being the first to examine both the composite sMMG IMF and its multi-resolution components concurrently, aiming to enhance our understanding of how these underlying components influence the composite IMF during exercise. In the present study, there was a significant decrease in the IMF from start to finish across all participants, regardless of sex. Although not directly tested in the present study, it has previously been suggested that decreases in the mean frequency of sMMG signal could potentially reflect fatigue-induced decreases in motor unit firing rates [10,40], the de-recruitment of fast-twitch muscle fibers [4,41], or both, which could reduce the global motor unit firing rate. Interestingly, the difference in spectral intensity shifts within the different frequency components revealed no change in the low-frequency content compared to the medium–high-frequency contents, which exhibited decreases. Even more interestingly, the greatest decrease occurred in the medium-frequency range. It is possible that patterns of response could be representative of several physiological factors, such as a greater or faster rate of fatigue for medium–high-threshold motor units, innervating fast-twitch fibers, compared to lower thresholds. However, interpretation specifically regarding motor unit behavior should be made with caution as these changes could potentially result from fatigue-related changes in factors such as intramuscular fluid pressure [38]. Nevertheless, these data reveal interesting characteristics of the underlying frequency components that make up the composite frequency most commonly reported in the literature. We encourage future research to similarly explore the mean frequencies in tandem with its multi-resolution constituent to better contextualize any shifts in the composite frequencies during exercise.

This study is not without its limitations. Firstly, no a priori power analysis was conducted, and the sample size was constrained by available resources [42], necessitating caution in the interpretation of our results. Additionally, while we chose to begin the analysis with the second wavelet and employed a window average for noise suppression, this does not invalidate other methodologies that may be equally suitable. It is also important to note that variability in the number of maximal voluntary isometric contractions (MVICs) performed and rest intervals between trials—ranging from 2 to 4 min—may have influenced performance outcomes. Future research should consider standardizing the number of MVIC trials and the length of rest intervals to minimize potential confounding effects. Finally, our findings are specific to the context of this study and should not be generalized to other forms of exercise. We urge caution in their interpretation and encourage further research to address these limitations and explore broader applicability.

## 4. Materials and Methods

### 4.1. Experimental Design

The changes in intensity within each wavelet domain were examined during a fatiguing, maximal, unilateral isometric leg extension in healthy, recreationally active men and women. Testing procedures involved performing isometric muscle actions on a modified leg extension machine (Body-Solid, GLCE365, Forest Park, IL, USA) equipped with a load cell (Honeywell Model 41 Precision Low Profile Load Cell, Charlotte, NC, USA). Neuromuscular responses were derived from sMMG signals recorded from the vastus lateralis of the non-dominant limb, with leg dominance determined based on participants’ kicking preference. The non-dominant leg was specifically selected for testing to standardize conditions across all participants, as it is typically less trained and less likely to have undergone specific conditioning compared to the dominant leg [43]. This approach was intended to minimize potential confounding factors related to prior training or habitual use.

**Figure 4:**
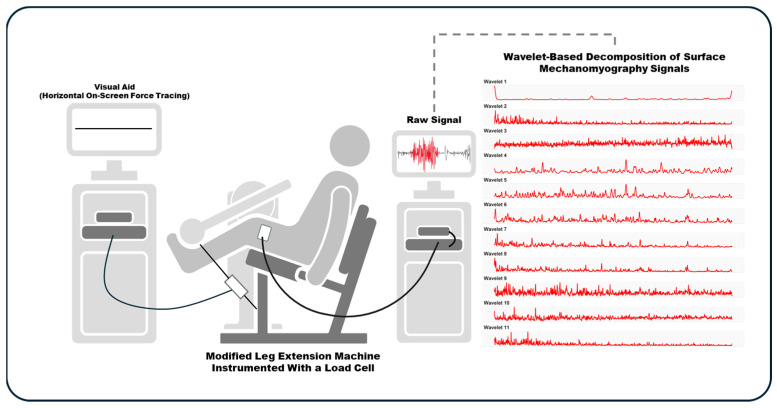
Overview of methodology. Neuromuscular responses were derived from surface mechanomyography (sMMG) signals recorded from the vastus lateralis of the non-dominant limb during a fatiguing, maximal, unilateral isometric leg extension. Following the fatiguing protocol, the intensities of the sMMG signal were resolved simultaneously in both time and frequency spaces using an sMMG-specific wavelet-based analysis.

This study was approved by the Institutional Review Board for Human Subjects (IRB #73699) and adhered to the ethical standards of the Helsinki Declaration. It is important to note that the data analyzed in this study are part of a larger investigation that included multiple dependent and independent variables [44,45], and the specific data presented here have not been previously published. A schematic diagram of the experimental setup is provided in Figure 4 for clarity and reference.

### 4.2. Participants

A sample of 21 recreationally active men (*n* = 11) and women (*n* = 10) between the ages of 18 and 35 years were utilized for this analysis. Specific participant characteristics can be found in Table 1. To be considered eligible for the present study, participants were required to have resistance training experience of at least 1 year and to have abstained from any strenuous physical activity for 48 h prior to testing. Additional exclusion criteria were based upon illness or any contraindications to physical activity identified using a health history questionnaire. All participants were informed of the risks and benefits of the study, completed a health history questionnaire, and signed a written informed consent document before participating in this study.

### 4.3. Fatiguing Protocol

The experimental session began with a standardized warm-up consisting of three submaximal unilateral isometric contractions (approximately 30%, 50%, and 80% of MVIC) of the leg extensors of the non-dominant limb at a joint angle of approximately 120° (where 180° corresponds to full extension). Participants then underwent an isometric strength assessment, which included 3 to 4 separate, 3 s maximal unilateral isometric leg extensions to determine maximal force. Each participant completed a minimum of three MVIC trials, with a fourth trial conducted only if there was an issue with one of the initial three. Consequently, no participant performed fewer than three or more than four MVICs. A rest interval of 2 to 4 min was provided between each MVIC trial to ensure recovery, and the trial resulting in the highest force was used as the reference MVIC for the fatiguing protocol.

Following the strength assessment, participants rested for 5 min before performing a sustained, maximal, unilateral isometric leg extension with their non-dominant limb until task failure. Task failure was defined as a 50% reduction in force relative to the reference MVIC. Throughout the fatiguing protocol, participants were provided with a visual aid in the form of a horizontal on-screen force tracing to help maintain their effort.

During the fatiguing trial, a single sMMG sensor (Entran EGAS FT 10, bandwidth 0 to 200 Hz, dimensions: 1.0 × 1.0 × 0.5 cm, mass 1.0 g, sensitivity 10 mV g^−1^) was used to record signals from the vastus lateralis of the non-dominant limb. Sensor placement was made in accordance with the recommendations from the Surface Electromyography for the non-invasive Assessment of Muscles (SENIAM) project (http://www.seniam.org, accessed on the 1 October 2021). Specifically, the sensor was placed at two-thirds of the distance from the anterior superior iliac spine to the lateral side of the patella. Finally, the sMMG signals were sampled at 1000 Hz using a 16-bit analog-to-digital converter (Model MP150, BIOPAC Systems, Inc., Santa Barbara, CA, USA).

### 4.4. Signal Processing

Initial signal processing began with manual segmentation, delineating the trial start at the achievement of the target force and thetrial end upon task failure. This procedure was performed using the Acqknowledge data acquisition software associated with the Biopac MP150 used during data collection.

Following segmentation, the intensities of the sMMG signal were resolved simultaneously in both the time and frequency spaces using an sMMG-specific wavelet-based analysis. Specifically, this analysis utilized specialized Cauchy wavelets, generated in accordance with Beck et al. [15], which can be characterized in frequency space by

(1) $$F\psi \left(f,f_{c},scale\right)={\left(\frac{f}{f_{c}}\right)}^{f_{c}\cdot scale}\cdot e\left(\frac{-f}{\left(f_{c}\right)}+1\right)f_{c}\cdot scale\;$$

where $F\psi \left(f,f_{c},scale\right)$ signifies the Fourier transform of the wavelet, *f* the frequency, $f_{c}$ the wavelet’s center frequency, and scale the scaling factor. The specific center frequencies were computed using the relation

(2) $$f_{c_{j}}=\frac{1}{scale}{\left(j+q\right)}^{r}$$

where $f_{c_{j}}$ is the center frequency for the *j*th wavelet, with parameters *q* and *r* optimally adjusting wavelet spacing in the frequency domain. Beck et al. [15] offers a more comprehensive description of the wavelet analysis and parameterization, which underpins our methodology.

This wavelet analysis generates an intensity matrix approximating the sMMG signal’s power, employing a filter bank of 11 wavelets (k = [0, 1, …, 10]), with center frequencies ranging from 2.07 to 118.63 Hz. However, the first wavelet (k = 0; $f_{c}$ = 2.07) was excluded to ensure that low-frequency content associated with factors such as movement artifacts was not included in the analysis.

The instantaneous mean frequency (*IMF*) of the time-dependent power spectral density was calculated as

(3) $$IMF\left(t\right)=\frac{f_{0}^{F}\omega P\left(t,\omega \right)d\omega }{f_{0}^{F}P\left(t,\omega \right)d\omega }$$

where *P*(*t*, *ω*) represents the time-dependent power spectral density, *ω* the frequency, and *F* the Nyquist frequency.

To reduce the impact of outliers, a one-second average was generated at both the start and end of each trial. This window was selected to minimize transient spikes in spectral intensity that may not represent the underlying physiological state of the muscle.

### 4.5. Statistical Analysis

All statistical analyses were performed in R (version 4.2.2; R Core Team, 2022, Vienna, Austria). Linear mixed effect models were all constructed using the lme4 and lmerTest packages and indices of model fit and model residuals were inspected using the performance and DHARMa packages. The code and data, including the signal processing code, used for analysis are available on the Open Science Framework (https://osf.io/z5vkp/?view_only=794d9e9fd5564efdb527da657d54545b (accessed on the 6 April 2024)).

To quantify the impact of sex on fatigability, our first analysis compared the time-to-task failure between men and women using an independent sample *t*-test. Next, linear mixed-effect modeling was used to delineate the interplay between sex and temporal dynamics of IMF. The resulting mixed-effects model, in Pinheiro–Bates–modified-Wilkinson–Rogers notation [46,47], was succinctly expressed as

IMF ~ Sex × Time + (1|Participant)(4)

where fixed effects, and interactions thereof, were included for sex and time, and time was operationalized as a continuous variable, with a coding scheme assigning a value of 0 at task initiation (‘Start’) and 1 upon task completion (‘End’).

In parallel, the sex-dependent evolution of log-transformed spectral intensity across wavelets was examined using a linear-mixed-effect framework. The model was parameterized as follows:

log(Intensity) ~ Sex × poly(Wavelet,2) × Time + (poly(Wavelet,2) + Time|Participant)(5)

where fixed effects, and interactions thereof, were included for sex, wavelet domain, and time; and both the time and wavelet domains were operationalized as continuous variables. Further, the log-transformation of spectral intensity effectively re-scales the measurements to operate on a relative scale, occupying the role of normalization in the process. Finally, to accurately encapsulate the relative change–frequency relationship, the wavelet domain was modeled as a second-order polynomial, which enabled the model to converge on a solution with normally distributed and homoscedastic residuals.

All linear mixed-effect models were fit with restricted maximum likelihood estimations, with random intercepts per participant to appropriately address the repeated-measure design. Maximal random effect structures were employed where feasible to refine the model accuracy and enhance generalizability. Finally, ANOVA tables were generated using the Type III sum of squares, complemented by Satterthwaite’s approximation for denominator degrees of freedom and the computation of F-statistics, thereby ensuring an accessible interpretation of the data. All effects and their variability are presented in unstandardized form [48], and for all inferential statistical procedures, the alpha level was maintained at 0.05.

## 5. Conclusions

The findings of this study revealed wavelet-specific changes in spectral intensity during fatiguing isometric contractions, with the most pronounced shifts occurring in the medium-frequency domains and trivial changes observed in lower-frequency domains. These results offer new insights into the complexity of neuromuscular signals during fatigue, providing a nuanced perspective beyond commonly reported composite measures such as mean frequency. Contrary to our hypotheses, no significant sex-based differences in time-to-task failure were observed. While previous research suggests that women are typically more fatigue-resistant than men during sustained isometric tasks, particularly at lower intensities, at high force levels, intramuscular pressure likely occludes blood flow in both sexes [36], potentially minimizing differences in oxygen delivery and fatigue resistance. These findings highlight the importance of task-specific factors in determining fatigue-related outcomes and highlight the potential of wavelet-based signal processing to uncover unique aspects of neuromuscular responses. Future research should investigate these signal dynamics across a wider variety of intensities, tasks, and exercise modalities to better understand the mechanisms underlying fatigue and their potential variability across different contexts. Incorporating direct physiological measurements, such as muscle oxygenation, may also provide further insight into the interplay between neuromuscular responses and fatigue during various forms of physical activity. Specifically, these analyses may help futher investigate exercise fatigability across diverse exercise conditions and musculoskeletal diseases, offering new opportunities to advance our understanding of neuromuscular adaptations and dysfunction.

## Figures and Tables

**Table 1:** Participant characteristics presented as mean ± standard deviation.

Measure	Men (*n* = 11)	Women (*n* = 10)
Age (yrs)	24.2 ± 2.9	23.5 ± 4.2
Height (cm)	179.2 ± 6.9	164.8 ± 6.3
Body Mass (kg)	83.4 ± 14.3	68.7 ± 13.8
Pre-MVIC Force (N)	969.2 ± 283.8	690.8 ± 221.6
Time-to-Task Failure (s)	43.9 ± 18.1	55.8 ± 17.7

Abbreviations: yrs = years, cm = centimeters, kg = kilograms, MVIC = maximal voluntary isometric contraction, N = newtons, s = seconds.

## Data Availability

The original data presented in the study are openly available in the Open Science Framework Repository at https://osf.io/z5vkp/?view_only=794d9e9fd5564efdb527da657d54545b (accessed on the 6 April 2024).
